# Contrasting cultures of emergency department care: a qualitative study of patients’ experiences of attending the emergency department for low back pain in the UK

**DOI:** 10.1136/bmjopen-2024-091158

**Published:** 2025-05-11

**Authors:** Clare Ryan, Catherine Pope, Lisa C Roberts

**Affiliations:** 1 University of Southampton, Southampton, UK; 2 Musculoskeletal Service, Hampshire and Isle of Wight Healthcare NHS Foundation Trust, Portsmouth, UK; 3 Nuffield Department of Primary Care Health Sciences, University of Oxford, Oxford, UK; 4 Therapy Services Department, University Hospital Southampton NHS Foundation Trust, Southampton, UK

**Keywords:** QUALITATIVE RESEARCH, ACCIDENT & EMERGENCY MEDICINE, Patient-Centered Care, Musculoskeletal disorders, Organisation of health services

## Abstract

**ABSTRACT:**

**Objectives:**

This article aimed to explore patients’ experiences of attending the emergency department (ED) for low back pain (LBP) and provides a theoretically informed analysis of the ED cultures perceived by patients to inform their experiences of care.

**Design:**

Multisite, cross-sectional qualitative interview study.

**Setting:**

Four NHS Emergency Departments located in the UK.

**Participants:**

47 adults (aged 23–79 years) who, in the past 6 weeks, had attended the ED for LBP (all types and durations). Purposive sampling was used to gain variation in the recruiting sites, and participants’ LBP and demographic characteristics.

**Interventions:**

Data were collected using individual, semistructured, telephone interviews (median 45 min duration) which were audio-recorded and transcribed verbatim. Analysis was informed using reflexive thematic analysis and ideal type analysis. Cycles of inductive and deductive analysis were undertaken, with Bourdieu’s concepts of field and habitus employed to help explain the findings.

**Results:**

We present three contrasting cultures of ED care for LBP, comprising (1) emergency screening only, (2) ‘cynicism and neglect’ and (3) appropriate and kind care. Taking each culture (field) in turn, we explore important differences in the content and delivery of care. Drawing on Bourdieu’s concepts of field and habitus, we consider the social and institutional norms and misrepresentations likely to underpin the thoughts and behaviours of ED staff (their habitus), and why these tended to vary based on where and by whom the patient was managed in the ED.

**Conclusions:**

Strategies to improve patients’ experience need to review the social and institutional norms that underpin staff habitus, the assumptions informing these norms and the voices that validate and reproduce them.

**ISRCTN registration number:**

ISRCTN77522923.

**STRENGTHS AND LIMITATIONS OF THIS STUDY**

- This article provides a theoretically rich analysis of the emergency department (ED) cultures perceived by patients to inform their experiences of attending for low back pain.
- People from underserved populations were purposively recruited to include those living in northern and coastal locations, socio-economically deprived areas, those identifying a minority ethnic background and people who lacked confidence in conversing in the English language.
- Although data were collected during the COVID-19 pandemic, the findings align with literature reporting people’s experiences of attending ED for any condition and remain salient in a postpandemic context.
- Although patient and public involvement was key to the study design and delivery, it was not undertaken during the main analysis, which may have furthered some insights.

## Introduction

 Low back pain (LBP) is a symptom rather than a disease and is characterised by pain between the 12th rib and the buttock crease.^1^ This condition affects most people at some point in their lives, and for the vast majority the cause is not serious.^2^ LBP can, however, be severe and/or disabling, particularly initially or if it persists, and is the leading cause of disability, both globally and in the UK.^3^ Clinical guidelines recommend that LBP is usually best managed in primary or community care. Following screening to exclude serious pathology, a biopsychosocial approach and supported self-management are recommended, with manual therapy and/or psychological therapies only used as part of a treatment package that includes exercise.^4^

Clinical guidelines recommend that ED care for LBP is only necessary for the small minority of presentations where the integrity of the spinal cord is threatened, or for serious medical conditions that masquerade as LBP.^5^ Globally, however, LBP accounts for around 4% of ED attendances.^6^ Based on 16.6 million UK ED attendances in 2021–2022 in the UK, this translates to around 50 000 ED attendances per month.^7^ Reducing ED demand, particularly those attendances that could be avoided, is a UK healthcare policy priority to ensure that ED resources are reserved for those who require this level of care.^8^

An emerging literature (comprising three single-site, qualitative studies, in the UK, Australia and the USA) has explored why people attend the ED for LBP, from the patient’s perspective.^9^,^11^ Key reasons for attending are reported to include the severe, disabling nature of pain and its impact on people’s ability to function; concerns about cause; perceived need for investigations; poor access to the general practitioner (GP) (or alternative provider) and following the advice of others, including healthcare professionals, to attend.

To understand how to best or alternatively manage those who attend the ED for LBP, it is important to understand how it is to receive care in the ED. Using these perspectives to inform the organisation and transformation of healthcare is recognised by the National Institute of Health and Care Research (NIHR) and NHS England to be fundamental to delivering services that are safe, effective and acceptable.^12^,^14^ The salience of the patient voice is particularly important where the services they attend differ from those recommended by policy and providers.

Several literature provide insight into how it is to attend the ED from different perspectives. The first of these explores patients’ experiences of attending the ED in general populations. This literature, synthesised in two mixed-methods systematic reviews and one qualitative evidence synthesis, comprises 21, 26 and 22 qualitative studies, respectively, with primary studies located in North America, Europe and Australia.^15^,^17^ Key issues identified to be important to the patients include how the ED waiting period is managed, how ED processes and outcomes of care are communicated to the patients and the nature of relationships between staff and patients, including how well empathy and compassion are enacted by staff. Graham *et al*^16^ further identify the importance to patients of their reasons for attending being taken seriously and of being involved in decisions about their care. These findings highlight that even when attending a service that provides emergency healthcare, the importance to patients of care being person-centred and aligned with patients’ perceived needs and priorities.

A second, related literature has explored patients’ experiences of being managed in the ED by a physiotherapist. This literature (comprising four qualitative studies located in Australia and one in the USA, with a total population of 145 patients)^1118^,^20^ is included due to the growing trend to integrate primary care staff including physiotherapists into the ED staff skill-mix, to help manage demand and optimise the management of low-acuity presentations. Key findings are that patients value being treated by a physiotherapist due to their expertise in managing musculoskeletal problems, particularly LBP; because they provided an alternative to pharmacological management of pain and due to physiotherapists’ proactiveness in arranging follow-up care. This work suggests the importance to patients of ED care that not only excludes serious deteriorating pathology and helps manage pain, but that also includes treatment options and facilitates access to follow-up care.

Finally, a body of ethnographic work, undertaken over the past six decades (in the UK, the USA, Europe and, more recently, Asia (including Hong Kong and Romania)), explores how ED care is delivered from the researcher’s perspective.^21^,^25^ These studies provide insight into socio-cultural aspects of ED care suggested to underpin individuals’ experiences. This literature argues that key relevant concepts include the primacy of the life-saving function of the ED; gatekeeping of ED resources (to align with the ED’s primary function); the moral evaluation of patients perceived by staff to have attended illegitimately (those who do not require this acuity of care) and reasonableness (circumstances that make the attendance reasonable if not clinically necessary). This literature highlights that the ED remit of providing life or limb-saving care is key to how staff deliver ED care.

Recognising the importance of managing LBP well, the policy priority of reducing clinically unnecessary ED attendances, the importance of the patients’ voice in informing the organisation and delivery of healthcare, and the apparent disparity between patients’ perceived needs and constructs that underpin the provision of ED care, this study aims to explore patients’ experiences of attending the ED for LBP.

## Methods

### Study design

This article is reported in line with Consolidated criteria for Reporting Qualitative research and Standards for Reporting Qualitative Research guidelines.^26 27^ The data were collected as part of a multisite, cross-sectional, qualitative interview study exploring why people attend the ED for LBP and patients’ experiences of attending. The findings related to why people attend the ED are reported separately.

This study was informed by the principles of interpretivism, an approach that seeks to understand how people interpret, understand and give meaning and significance to the social world, and which recognises that how people interpret their situation is informed by their individual context. This approach further recognises that the researcher’s values are inherent to and inseparable from the research process, and that findings are a co-construction, representing the researcher’s interpretation of participants’ perspectives.^28^ Aligning with the position of the NIHR^14^ and NHS England,^8^ we perceive the importance of exploring patients’ perspectives and experiences of healthcare, recognising that such inquiry can surface hidden agendas in healthcare and safety issues, and provide new insight into how services might be developed to better align with patients’ perceived needs and priorities.

The lead author, CR, was a white British female doctoral candidate and spinal clinical specialist physiotherapist (working in a community NHS Trust). CR had previous experience and training in qualitative inquiry from two masters’ research projects and doctoral training. Other members of the research team comprised a professor in medical sociology and a clinical professor and consultant musculoskeletal physiotherapist.

### Setting, participants and recruitment

This study was located in the UK, with participants recruited from the EDs of four English NHS Trusts. Recruiting sites were selected to include locations not commonly included in research, and sites likely to enable a sample with variation in ethnic and socio-economic background and healthcare experiences. Sites also included those that were and were not regional spinal centres and those that did and did not include physiotherapists in the staff skill-mix. This latter criterion was included to reflect physiotherapists’ roles within the ED.

Participants were adults (aged ≥18 years) who had attended the ED in the past 6 weeks for LBP, who had capacity to consent and were unknown to the researcher. Aligning with the population who present to the ED, as identified in the literature and during site visits,^29 30^ we included people with all types and durations of LBP (including those for whom a non-musculoskeletal cause for LBP had been identified in the ED). Participants were sampled purposively to gain variation in age, ethnicity (using the National Office for Statistics classification of ethnicity), socio-economic status (based on the Index of Multiple Deprivation by Postcode),^31^ LBP characteristics (including the history of LBP, related healthcare use and the likely clinical acuity of the presentation) and the ED attended. The likely acuity and nature of participants’ presentations were inferred by the research team from participants’ accounts (no information was sought from the ED team relating to participants’ presentations or management). To help achieve variation in the sample, a sampling grid detailing sought-after characteristics was employed. Progress in achieving variation was monitored throughout recruitment, and once half of the required sample had been recruited, sites were asked to preferentially and then exclusively recruit for characteristics, including having an ethnicity other than white British, serious pathology and recruitment from the lowest recruiting site. No minimum threshold was applied. Accessing participants with diverse LBP and socio-demographic characteristics, including those from underserved populations, provides the potential for credible, transferable findings.^32 33^

Potential participants were identified and approached by their treating clinician or a research nurse, either at the time of their ED visit or by telephone shortly following this. Those interested were provided with the study information pack, including the participant information sheet, and information about how to identify to the researcher their interest in participating. We recruited at the four sites concurrently and included people who attended at all times of day and night, and those who had were admitted to hospital from the ED. We aimed to recruit up to 50 participants to align with our aims to achieve a varied sample, undertake in-depth analysis and be confident in our conceptualisation of the data.^32 33^ 81 potential participants were approached, 75 provided consent for their details to be forwarded to CR and 47 consented to participate. Interviews were arranged at the participant’s earliest convenience. Express written consent to participate was gained at the time of the interview.

### Data collection

Data were collected using individual, semistructured, telephone interviews (Microsoft Teams audio-calls), undertaken between August and December 2021 by CR. Interviews occurred during the UK COVID-19 pandemic when social distancing and travel restrictions remained in place and many outpatient healthcare appointments were undertaken remotely, usually by telephone. At the start of data collection, an internal pilot was completed with three participants who met the inclusion criteria, and as the content, topic guide and key wording of questions were not altered significantly, the findings were included in the analysis. Although a topic guide was used (online supplemental material 1), participants were encouraged to speak at length and questions focused on participants’ expectations of care, whether care aligned with perceived need and if in similar future situations patients would make the same decision. Most interviews included only the researcher and participant; however, two interviews were undertaken through an interpreter, and three participants attended the interview with their partner, either to help with language issues or aid their memory. Interviews were continued until the maximum variation sample had been achieved, with the 47 interviews providing the breadth and depth of data sought. Immediately following the interview participants were asked to self-report their socio-demographic characteristics.

Interviews were audio-recorded, transcribed verbatim by a professional transcriptionist and verified by CR. Field notes were made during and immediately following the interview (to guide subsequent interviews and the initial analysis). Participant validation of the transcripts or findings was not undertaken as the usefulness of this strategy is contested.^34^ Monthly team meetings were used to discuss recruitment and sampling, to make sense of analytical impressions and consider the position of the researcher.

### Patient and public involvement

As part of the original study, 22 patient and public representatives and 2 patient and public involvement and engagement (PPIE) leads were involved in designing this study. The representatives helped prioritise and shape the research questions, and refine the methods and the issues to be explored at interview. Two PPIE representatives helped develop patient-facing materials (study letter; participant information sheet; consent form) to refine the interview topic guide and pilot remote interview technology.

### Data analysis

Data were initially analysed thematically, informed by Braun and Clarke’s Reflexive Thematic Analysis,^35 36^ with this approach selected to align with the interpretivist aims of the study. The analysis was undertaken by CR, principally following rather than alongside data collection due to the speed of recruitment. After initial familiarisation with the data, CR coded the data set inductively, including open coding and coding for processes, interactions, outcomes, conflicts, meanings and emotions. Initial themes were then generated, developed and refined. Having identified different ‘types’ of ED experience, our analysis then drew on Ideal Type Analysis, a qualitative research approach used to help develop typologies.^37 38^ Here the analysis included constructing the ‘types’ of experience (‘ideal types’); identifying the cases that best evidenced these types (‘optimal cases’); forming the ideal type descriptions and checking credibility and making comparisons.

Our analysis was also theoretically informed by Bourdieu’s concepts of field and habitus.^39^ These concepts were used as deductive codes, and we created analytical memos to consider how our data aligned with Bourdieu’s ideas. We iteratively considered the credibility of our emerging explanation, searching for detail, variation, complexity, disconfirming cases and alternative explanations.

### Bourdieu’s concepts of field and habitus

Bourdieu uses the concept of field to explain that in society individuals interact in social arenas with a common purpose, worldview and codes of behaviour.^39^ For individuals to be accepted and have status in a particular field, their beliefs, values and behaviour, or habitus, are required to align with those expected of the field, with deviance from this adversely affecting people’s status within the field. These worldviews and codes of behaviour are informed by social and institutional norms that are upheld by those with elevated status and for whom these norms are often advantageous. While structural issues such as privilege are recognised by Bourdieu to be key to people’s capability to align with the worldview and codes in a given field, the impact of such issues may not be recognised or may be misrepresented within the field. Insurgents (those who bring new ways of thinking) are accepted only when they are perceived to offer resources that are advantageous to the field.

## Findings

### Setting and sample characteristics

The recruiting sites selected comprised two EDs in the north and two in the south of England. The hospitals in which the EDs were located ranged in size from 850 to 1300 inpatient beds and included populations in rural, coastal and urban areas. Two sites were regional spinal centres. Three of the four sites included physiotherapists in the staff skill-mix, with physiotherapists managing patients autonomously, seeking medical help if indicated. Patients were recruited from both ED majors and minors (terms which refer to high-acuity and low-acuity treatment areas within the ED), with majors led by a doctor and minors by a nurse, with oversight from a doctor. Physiotherapists worked in both types of treatment area, but predominantly minors.

The data set comprised interviews with 47 participants, with interviews lasting an average (median) of 45 min (23–156 min). Participants (21 females and 26 males) ranged in age from 23 to 79 years (median 42 years), and 58 ED attendances were discussed. Eight participants attended the ED more than once for this episode of symptoms; six participants attended twice, one three times and one four times. The numbers from each recruiting site comprised 11, 9, 22 and 5 participants, respectively.

Sample characteristics are detailed in Box 1, and individual participant details are presented in online supplemental material 2. Variation was achieved in age, sex, geographic location and the nature of LBP attended for. Relating to underserved populations, all participants were recruited from northern or coastal populations; 10 participants had an ethnicity other than white British, including 2 who took part with the aid of an interpreter; and 21 participants lived in postcodes in the four most socio-economically deprived deciles.

Box 1 **Sample characteristics**

- **Ethnicity:** 37 participants were white British, 3 were white other; 3 Asian British; 2 mixed ethnic groups; 1 black British or African and 1 participant’s ethnicity was other.
- **Socio-economic profile:** Based on the Index of Multiple Deprivation (by postcode), 21 participants lived in the lowest 4 deciles (including 7 in the lowest decile), 23 participants in the highest 4 deciles and 3 participants in deciles 5 and 6.
- **Employment status:** 35 participants were in paid employment; 6 were retired, 1 was a homemaker and 5 were unemployed.
- **LBP presentation:** Based on participants’ descriptions, 16 participants presented with non-specific LBP; 22 had radicular or neurological symptoms (14 of whom had symptoms consistent with suspected Cauda Equina Syndrome); 7 had a visceral presentation; 2 participants had both non-specific and visceral LBP; and 2 had serious pathology (both vertebral fractures, one traumatic and one osteoporotic).
- **Symptom duration this episode:** At the time of attending ED, the median duration of LBP symptoms was 4 days (range less than 24 hours to 3 months).
- **Healthcare prior to attending ED:** 41 participants had attempted and 39 achieved contact with a health professional other than ED provider prior to attending. This included 23 who had attempted or achieved contact with the GP and 18 with NHS 111. 29 participants had been advised to attend by a healthcare professional or triage service, or ED had been suggested to be ‘an option’.
- **Ambulance use**: 12 participants arrived by ambulance.
- **Multiple attendances:** 8 participants attended ED more than once for this episode of symptoms.
- **LBP history**: For 12 participants, this was their first episode of LBP. Of the remaining 35 participants, 24 participants had previously experienced moderate or severe LBP, and 11 had previously experienced low-level LBP.
- **Previous healthcare use for LBP:** 27 participants had previously accessed healthcare for LBP. 12 had attended ED for LBP during a previous episode of symptoms.

ED, emergency department; GP, general practitioner; LBP, low back pain; NHS, National Health Service.

### Summary

We present a theoretically informed analysis of the three contrasting cultures perceived by patients to inform their experiences of ED care for LBP, comprising (1) emergency screening only, (2) ‘cynicism and neglect’ and (3) appropriate and kind care. Drawing on Bourdieu’s theories,^39^ we consider how the thoughts and behaviours, or habitus, of ED staff reflect perceived social and institutional norms, which differ according to where and by whom the patient was managed in the ED.

### A culture of emergency screening only

The first culture, one of emergency screening only, was equally likely to be experienced by those who attended ED majors or minors (high-acuity and low-acuity treatment areas within the ED); those who were or were not managed in regional spinal centres and those who were treated by doctors, nurses or physiotherapists. In this culture, patients received a brief clinical assessment to exclude a biomedical emergency and were advised to contact their GP to address any additional needs. This is perhaps the ED culture that might be expected and one that aligns with an ED remit of providing critical or life-threatening or limb-threatening care. Notably, in this culture, there was little mention of staff attitude or manner (either positive or negative), with the key characteristic of the field being care that was limited to an emergency screening.

As evident in the data extract below, patients perceived that receiving care in this field had been ‘a waste of time’, because although a serious cause had been excluded and pain relief received, their perceived needs relating to diagnosis, investigations, prognosis and treatment had not been addressed.

I never got any follow-up, I've not had a scan, I'm still in as much pain. To be quite honest, except for getting some painkillers which will be running out any day now anyway … it was just a waste of everyone’s time … I wasn't advised on what was wrong with me or how long it would take to get better or how to treat it. (P36)

Patients did, however, recognise why ED resources were prioritised for those with emergency presentations, even when this limited the care available to them.

I think they were happy that nothing was bleeding, nothing was protruding, just get me drugged up and send me home … And they can't waste the resources … I mean they've got a young lad in on a stretcher under a blanket fully clothed, eyes completely closed, it looks like he’s on death’s door. I guess everyone that comes in with a bad back or a sore finger or whatever, you can't go 'Get them in the X-ray room, let’s have a look'. But as a selfish person, human being, you always want to know. (P31)

While patients reported being advised to contact their GP or community services on discharge from the ED, to gain help to manage their LBP symptoms, for many, poor access to the GP had informed the decision to attend. Although poor GP access was acknowledged to be an issue by ED staff, it was not accepted as a reasonable reason for attending, nor a reason to extend ED care beyond an emergency screening.

Following the ED attendance, difficulty accessing follow-up care from the GP or a pharmacy, for example, to discharge a pain relief prescription, was also reported to be an issue.

The pharmacist said, 'He’s going to have to go to his doctor’ … and my partner said 'Why do you think I'm here? He can't move. He can't just walk into his doctor’s'. (P31)

We argue that in this culture the worldview and habitus of ED staff reflect the primacy of the ED’s life-saving function, with gatekeeping of ED resources (limiting care to an emergency screening) perceived by staff to be necessary and acceptable to facilitate the functioning of the ED and to preserve resources for those with high-acuity presentations. Examples of these resources include staff time to undertake a thorough examination and discuss the diagnosis, prognosis and optimal management, and to refer on or advise patients how to access follow-up care. Issues other than biomedical acuity, such as poor access to the GP or difficulty accessing medication following attending the ED, were not perceived by staff to be a reason to extend ED care beyond an emergency screening.

### A culture of ‘cynicism and neglect’

The second culture identified, using an in vivo code, was one of ‘cynicism and neglect’. This tended to be more likely to occur in majors (high-acuity treatment area); in EDs located in regional spinal centres and by those treated by doctors or nurses rather than physiotherapists. Here, not only was care limited to an emergency screening, but ED staff were perceived to cynically presume that patients had attended unnecessarily. This was perceived to result in care that was cursory and neglectful because it failed to address the patient’s urgent need to exclude a serious cause, receive a diagnosis, gain control of pain or access the help necessary to regain the ability to function.

They cannot just look at you and ask you a few questions and then send you home. That is so inhuman … Take the time that is necessary to go through the procedures … I know there are people who might be abusing the system …. but then there are also people who go there for genuine reasons. (P46)

Notably, participant 46 subsequently underwent emergency surgery.

It was evident from participants’ accounts that care in this culture was perceived to be inattentive, incomplete, focused on rapidly achieving discharge and that at times this resulted in carelessness. This approach to care was perceived by patients to reflect staff cynicism about the legitimacy of their presentation.

The male nurse practitioner came in and started pulling my jacket on and getting my stuff together, really quickly; they were trying to get rid of me. And I said, 'What about my ECG and my blood results, are they OK?' And he was like 'Yeah they'll be OK' …. [I got to the] front doors of A&E and suddenly realised when they've booted me out so quickly that they'd left the cannula in my arm'. (P41)

How it felt to receive care in this type of culture was a key issue, with participants using terms such as a ‘meat factory’ and ‘inhumane’ to describe this.

The hospital is a bit of a meat factory …I was shoved in and shoved out of the other end. (P1)

These terms suggest that care was perceived to be delivered in a way that was procedural, lacking in compassion and that the content of care did little to reduce the impact and duration of suffering. Key issues included pain being poorly managed throughout the ED attendance, particularly during the protracted waiting period, and failing to provide patients with support to mobilise when in severe disabling pain, or a discharge plan that addressed patients’ needs.

I couldn't even walk to the door to meet my partner; I was in that much pain. And somebody in the corridor on my way out had to go and get me water so I could take some more tablets basically just to get me out of the hospital, I couldn't even stand up. And I was sent away like that because it wasn't cauda equina. The phrase she used was 'It wasn't a medical emergency' so therefore you're going. (P47)

I just feel like both times I've been there it’s been like … 'Oh if it’s broken, we'll help you but if it’s not we won't' … I'm worried that this back injury is really going to set me back. And I haven't really had any advice about what to do in the future for post-treatment for it. (P2)

Furthermore, staff were occasionally reported to infer that patients exaggerated their symptoms. The two extracts below, from P41, provide an example of this, with P41’s presentation initially presumed to be illegitimate.

He [ED doctor] launched into this speech 'I am the gatekeeper of the hospital … Nurses have reported to us you haven't been writhing around in pain. So why have you come here?' … ‘And it’s all mental illness anyway, I've looked at your MRI result and I can see that you've got a herniation but it’s all mental illness. You do know that don't you? (P41)

He used this prong thing on the bottom of my feet. And because it wasn't reacting on one side he said, 'Right so you're going to be admitted to the A&E ward’, it was just so bizarre. How he completely changed his tack. (P41)

The outcome of care in a culture of ‘cynicism and neglect’ was distress: patients perceived their reasons for attending had been invalidated, had little confidence that an emergency presentation had been excluded and had lost faith that the health system was willing or able to address their needs, within or beyond the ED.

And I remember my wife coming and picking me up and then driving home and me thinking, where is this going to leave me now? If this is what I'm getting from the hospital, then what is going to happen to me? And that is that mental agony that I was feeling. (P46)

I was just traumatised really with the thought that I had to carry on with this pain the way I had been … it was just awful. (P47)

It was then the weekend again and I'd run out of codeine and my partner was trying everywhere. He must have made about ten calls to 111 to try and get some codeine just for over the weekend. (P47)

We suggest the worldview of this ED culture again reflects the primacy of the ED’s life-saving function, with gatekeeping of ED resources perceived by staff to be necessary and acceptable. However, the way this worldview was enacted (staff habitus), with patient’s distress, physical needs and motivations for attending being ignored or called into question, resulted in perceptions of cynicism, moral evaluation and neglect.

It is perhaps unsurprising that this type of culture tended to be more likely to occur in ED majors, where ‘clinically unnecessary’ presentations may be perceived by ED staff to divert care away from those with high-acuity presentations. However, we note that patients do not choose to attend majors but are instead triaged there by ED staff. Moreover, patients managed in majors, including patients who were perceived to have attended illegitimately, had often been advised to attend the ED by a healthcare professional. Furthermore, gatekeeping ED resources based on the perception that patients should access healthcare using recommended healthcare service ignores the barriers to access that are experienced disproportionately by underserved populations.^40^ We argue that in this culture staff misattribute patients’ ED attendance to be an issue of choice and individual responsibility.

### A culture of appropriate and kind care

The third ED culture reported by participants was one of appropriate and kind care. This culture was more often experienced when patients were either managed as an emergency presentation in majors, or as a less urgent presentation in minors (a low-acuity treatment area) and by a physiotherapist. In this culture, in sharp contrast to a culture of ‘cynicism and neglect’ there was concordance between the patient and clinician about the potential of the consultation, with the content and delivery of ED care aligning with patients’ perceived needs.

In this culture, pain and its adverse impact on mobility were recognised, validated and proactively managed.

The triage nurse came out to me. When she saw that I was struggling, she held my arm and helped me into the triage room … She asked me if I'd taken any medication … gave me some co-codamol tablets and then she went and got me a wheelchair. (P42)

She understood how I felt, and she obviously saw I was in tears and how much pain I was in. She obviously made sure that I was given pain relief, and she did organise me a bed to lay down. (P22)

Care was reported to be delivered with compassion, empathy and respect.

The nurses and the physio that examined me, just the empathy of them. They could see I was in a lot of pain, and I was struggling. And they just couldn't do enough for me. The physio actually said at one point, … he went through something similar and his empathy levels with his patients shot up. And that really came through, the fact that he knew what I was going through. (P42)

All the tests that he went through explaining everything thoroughly. Why he needed to do it, what he was doing, he let me do it in my own time. I just felt like he cared really. (P43)

It was also evident that in this culture help was provided to ease health system issues both within and beyond the ED. Examples of this included ED staff collecting medication from the pharmacy, saving patients a long walk while in pain; and ensuring that on discharge patients had a detailed, individualised plan of how to manage their pain and access further help. The outcome of a culture of appropriate and kind care was that patients perceived that their needs had been attended to and that there was a plan to manage their LBP going forwards.

It just felt like everything was going to be sorted. (P43)

We suggest that in this ED culture, in addition to providing ethical, dignified care, the worldview and habitus of ED staff align with the concepts of person-centred care, therapeutic alliance and a biopsychosocial model of health.^41^,^45^ Patients who experienced this culture were more likely to be managed by a physiotherapist precisely because contemporary physiotherapy training emphasises the importance of adopting a biopsychosocial, person-centred approach, and physiotherapists have expertise in managing musculoskeletal conditions, including LBP. Furthermore, physiotherapists often work autonomously and are therefore less likely to feel pressure to adopt an emergency medicine worldview. Moreover, in the UK, primary care staff (including GPs and physiotherapists) have been integrated into, or alongside, the ED staff skill-mix specifically to manage those with less urgent presentations using a primary care rather than an emergency medicine approach (to preserve ED resources).^46^ These issues create the situation whereby physiotherapists working in the ED can adopt a biopsychosocial approach, with their expertise in managing LBP providing the cultural capital necessary for this approach to be accepted by other staff. We further suggest the relevance of this culture being more likely to exist in minors, a low-acuity treatment area which is usually led by ED nurses: innovative practice in this setting does not pose a threat to the morbidity or mortality of other patients, nor to the ED staff hierarchy.

## Discussion

In this study, we explored patients’ experiences of attending the ED for LBP and presented a theoretically informed analysis of the cultures perceived by patients to inform their experiences of ED care for LBP. These cultures comprised (1) emergency screening only, (2) ‘cynicism and neglect’ and (3) appropriate and kind care. Drawing on Bourdieu’s concepts of field and habitus,^39^ we identified social and institutional norms likely to underpin the thoughts and behaviours of ED staff and why these differed between the cultures.

Our finding of the importance of culture to patients’ experiences of ED care for LBP aligns with the definition of the Beryl Institute, a global patient experience community, which recognises organisational culture to be integral to patient experience.^47^ Furthermore, our findings build on recognition in the literature that concepts, including the primacy of the life-saving function of the ED, legitimacy and gatekeeping, help explain how ED care is delivered. Moreover, our findings of a culture of appropriate and kind care that tended to be more likely to be experienced by those treated in ED minors (a low-acuity treatment area) and delivered by a physiotherapist align with the findings of Naylor *et al*’s^48^ mixed-methods study that explored person-centredness among physiotherapists working in a UK ED (including 26 surveys and 11 in-depth interviews). Naylor *et al* highlighted the importance to physiotherapists working in the ED of ‘entering the patient’s world’ and showing empathy for patients’ sense of desperation; their recognition that this approach was in contrast to the dominant biomedical model, that it was not their place to judge patients for having attended for low-acuity presentations and the need to provide a plan to help patients manage their problem beyond the ED. Our analysis builds on these literature by presenting three contrasting cultures of ED care and identifying how staff thought and behaved towards patients (their habitus) was informed by social and institutional norms that varied based on whether the patient was treated in minors or majors and the profession of the staff member. Our findings therefore suggest that strategies to improve patients’ experiences of ED care for LBP should consider ED social and institutional norms and the voices that validate and reproduce these.

Recognising the conflict between an ED remit that emphasises life or limb-threatening care and LBP guidelines that recommend a person-centred biopsychosocial approach,^4 5^ our analysis suggests the need for stakeholders (including patients and staff from different professional backgrounds) to work out how to best navigate this tension (in both major and minor settings). Recognising that those who attend the ED are more likely to experience issues of health inequity, such as poor access to primary care, socio-economic deprivation and comorbid physical and health conditions,^40^ we emphasise the importance of ED care that aligns with best practice guidance so that it does not perpetuate health inequity and poor health outcomes. Our findings suggest the importance of care that is complete and that clearly signposts access to follow-up care (including information about medication, self-management and how and from where patients can access clinical review).

Our findings also highlight the importance of ED staff being alert to and willing to disrupt ED social and institutional norms that result in cultures of cynicism and neglect, particularly in ED majors and regional spinal centres. Furthermore, we suggest that UK policy and media campaigns that emphasise individual choice and responsibility when deciding where to attend the ED, such as the ‘choose well’ campaign,^49 50^ fail to reflect (or misattribute) how issues of health inequity inform the decision to attend the ED, and are likely to perpetuate and sanction such cultures. We therefore suggest that healthcare policy should instead prioritise enabling urgent access to excellent primary and community care. Moreover, we argue the relevance of structural competency training for those who shape healthcare policy, ED commissioners and staff.^51^

Finally, our findings suggest the potential for a ‘preferentially minors’, biopsychosocial approach to managing LBP in the ED, and the value of including physiotherapists in the skill-mix. We emphasise the relevance of such an approach being more likely to be accepted if undertaken in minors (a low-acuity treatment area) and by those with expertise in managing LBP, and who therefore have the cultural capital necessary to facilitate the adoption of innovative care. Further research is needed to evaluate the safety, effect, feasibility and patient and staff acceptability of this approach.

Key strengths of this study include the sampling strategy and recruitment methods used, which enable the voices of underserved populations to be included, and using theory to help to explain our findings. Using theory increases the explanatory reach of the findings and informs strategies to address this situation. One limitation is that we did not include PPIE involvement during analysis, and this may have furthered insight. Furthermore, additional insights could have been gained if the ED attendances were observed; however, this was not possible. Finally, although data were collected during the COVID-19 pandemic, our findings align with existing ethnographic work^21^,^25^ and issues evident in the population level literature^15^,^17^ and remain salient in a postpandemic context.

## Conclusions

This article explores patients’ experiences of attending the ED for LBP and presents a theoretically informed analysis of the ED cultures perceived by patients to inform their experiences of care. We delineated three contrasting cultures of care, comprising (1) emergency screening only, (2) ‘cynicism and neglect’ and (3) appropriate and kind care, noting important differences between these cultures relating to the content and delivery of care and where and by whom the patient was managed in the ED. Drawing on Bourdieu’s concepts of field and habitus, we identified social and institutional norms likely to underpin the thoughts and behaviours of ED staff and why these differed between the cultures. Strategies to improve patients’ experience of ED care for LBP, and reduce unwarranted variation in care, should recognise the social and institutional norms that underpin how staff think and behave when managing LBP in the ED, the assumptions that underpin these norms and the voices that validate and reproduce them.

## Supplementary material

10.1136/bmjopen-2024-091158 online supplemental material 1

10.1136/bmjopen-2024-091158 online supplemental material 2

## Data Availability

No data are available.
