# Supplementary material for: Contrasting cultures of emergency department care: a qualitative study of patients’ experiences of attending the emergency department for low back pain in the UK
Source: BMJ Open. 2025 May 11;15(5):e091158. doi: 10.1136/bmjopen-2024-091158 (PMC12067803; doi:10.1136/bmjopen-2024-091158)
Supplement: online supplemental material 1 [file bmjopen-15-5-s001.docx]

**SUPPLEMENTARY MATERIAL 1 TOPIC GUIDE**

**Tell me about what you hoped and expected to happen when you attended ED**

- Diagnosis
- Specific processes that you wanted to occur & why?
- Longer term mx beyond ED
- What you wanted to achieve by attending & why?
- What would have been a good outcome?
- What did you expect to happen, why and what were your thoughts about this/ would this have met your needs?

**Explain what it was like to attend ED for LBP**

- What happened when you were there? (who you saw, what they did, diagnosis, mx, how long there)
- How felt about being there and what happened?
- What went well & what could have gone better?
- On a personal/human level how was the overall care you received?
- What the environment was like for someone attending for low back pain
- How well did it fit around your other commitments/ circumstances?
- The agenda of attending ED

**Describe what you got out of attending**

- How do you feel about the problem now that you’ve attended?
- How useful was it to attend?
- The extent to which your needs were met?
- Understanding of problem; prognosis; treatment options; follow up; what to do in a similar future situation?
- Any resources e.g., information/ contacts/referrals
- Your thoughts or feelings about having attended.
- Consistency in messages in ED/ between ED & other HCP about the diagnosis & required mx?

**Tell me about how things are now with your back/the problem**

- Impact of problem.
- Understanding of nature of problem; prognosis; treatment; self-management; next steps; what to do if not improving/worsens.
- Access to/ plans for further healthcare/ referrals.
- How are things different for you having attended ED?
- Planned next steps: self-care; GP; other healthcare; repeat ED visit
- What about this condition will indicate to you things are getting better or worse: symptoms & function? (what would you be worried about/pleased about?)

**Explain what you would do in the same/similar situation in the future and why**

- Would you seek healthcare?
- Would you attend ED?

**Impact of covid**
