# Supplementary material for: Contrasting cultures of emergency department care: a qualitative study of patients’ experiences of attending the emergency department for low back pain in the UK
Source: BMJ Open. 2025 May 11;15(5):e091158. doi: 10.1136/bmjopen-2024-091158 (PMC12067803; doi:10.1136/bmjopen-2024-091158)
Supplement: online supplemental material 2 [file bmjopen-15-5-s002.docx]

Supplementary Material 2: Individual Participant Characteristics

**Key**

LBP low back pain

MSK Musculoskeletal

sCES suspected Cauda Equina Syndrome

| **No.** | Age years | Gen  der | LBP duration  before ED | LBP history & treatment | LBP healthcare before ED | HCP advice to attend | >1 ED visit for LBP this episode | Previous ED visit for LBP | Transport to ED | Likely nature of LBP |
| --- | --- | --- | --- | --- | --- | --- | --- | --- | --- | --- |
| 1 | Late 40s | M | 2-3 weeks | 4-year history episodic LBP | GP tel consult | Yes: GP | No | No | Lift | MSK LBP, sCES & radicular symptoms |
| 2 | Late 30s | F | 12 hrs | Long term history recurrent LBP | Nil | No | No | No | Lift | MSK non-specific LBP following trauma (fall) |
| 3 | Late 20s | M | 3 weeks | 12-year history recurrent LBP | Physio | Yes: Physio | No | Yes | Lift | MSK LBP, sCES & radicular symptoms |
| 4 | Mid 30s | F | 7 weeks, acute flare that day | Previously only very mild symptoms | GP, urology, 111 & A&E | Yes: GP | Yes | No | Lift | Non MSK LBP: kidney stones |
| 5 | Late 40s | M | 2 days, acute flare that day | Long history low level and recurrently severe LBP | 111 | No | No | Yes | Lift | MSK non-specific LBP |
| 6 | Early 40s | M | 2 days, acute flare that day | 10 months LBP low level worsening over time | 111 | Yes: 111 | No | No | Ambulance | Non-specific LBP or visceral (unclear) |
| 7 | Late 40s | F | 2 weeks | One previous episode 2 years ago. NHS physio | GP | No | Yes | No | Lift/ taxi | MSK non-specific LBP and referred leg pain |
| 8 | Early 40s | F | 3 weeks | Nil previous | GP tel consult | No | No | No | Lift | MSK non-specific LBP |
| 9 | Early 40s | M | That day | 13-year history including fracture & discectomy | Nurse tel consult at spinal surgery clinic | No | No | Yes | Ambulance | MSK LBP & radicular symptoms 3 weeks post discectomy |
| 10 | Early 20s | F | 2 days before first visit | Long history mod LBP. One previous severe episode | 111 before each of ED visits | Yes: 111 | Yes | Yes | Lift/ ambulance | MSK LBP, neurological symptoms sCES |
| 11 | Mid 20s | F | 1-week escalating pain | One similar episode 10 years previously | GP x2 & 111 | Suggested by GP | No | Yes | Taxi | Non- MSK LBP, ?Gynae cause |
| 12 | Early 30s | F | 2-3 days | Nil | 999/111 (unclear, partner made call) | Yes: 999/111 | No | No | Lift | MSK non-specific LBP |
| 13 | Mid 30s | M | This episode that day | One year history of recurrent flares | 111 | No | No | No | Lift | MSK LBP and radiculopathy |
| 14 | Late 20s | F | 2.5 days | Nil | 111 | Suggested by 111 | No | No | Lift | Non-MSK ?gastroenteritis |
| 15 | Mid 70s | F | That day | Spinal fracture managed surgically | GP | Yes: 999 | No | Yes | Ambulance | MSK non-specific LBP |
| 16 | Mid 40s | M | That day | Nil | Nil | No | No | No | N/A | MSK non-specific LBP |
| 17 | Mid 50s | F | 1 month | This episode 6 months. Previous diagnosis of CES. Plus, one additional flare. | GP, referred to spinal specialist | Yes: spinal clinic (triaged from referral) | No | Yes | Drove self | MSK LBP, radicular symptoms, sCES |
| 18 | Mid 60s | F | That day | One previous episode quickly resolved with no treatment | GP tel consult | Yes: GP | No | No | Lift | MSK LBP & neurological symptoms and sCES |
| 19 | Late 20s | M | 3 months | Nil | GP tel consult. Private physio. NHS physio tel consult | No | No | No | Taxi | MSK LBP, neurological symptoms, sCES |
| 20 | Mid 70s | F | That day | Low level intermittent symptoms managed by chiropractor | 999 | No | No | Yes | Ambulance | MSK LBP traumatic vertebral fracture |
| 21 | Mid 40s | M | 10 days | Discectomy 12-14 years ago. Nil since. | GP E-consult & tel consult. Chiropractor. GP referred to spinal clinic | No | No | No | Lift | MSK, radicular pain & sCES symptoms |
| 22 | Mid 40s | F | 1-week sCES | 17 months ongoing LBP symptoms | GP; MRI; Epidural. Rheumatology 111; OOH GP | Yes | Yes | Yes | Ambulance | MSK, radicular symptoms, sCES |
| 23 | Early 30s | F | 4 days | One previous episode with MRI & specialist opinion | Nil | No | No | No | Lift | MSK non-specific LBP |
| 24 | Early 70s | M | 4 days | One previous severe episode 35years ago. | Nil | No | No | No | Taxi | MSK LBP & back related leg pain |
| 25 | Mid 20s | F | That day | Nil | Nil | Yes: 111 | No | no | Taxi | LBP post trauma associated with abdominal pain |
| 26 | Early 60s | M | 10 days neurological symptoms  2.5 weeks LBP | Long history of recurrent LBP & sciatica. One previous ED visit. Physio, chiropractor, MRI, PPT | Physio tel consult. | Yes: physio | No | Yes | Lift | sCES, MSK LBP & neurological symptoms |
| 27 | Early 30s | M | 3 weeks | Nil | GP | Yes: GP | No | No | Lift | MSK LBP with neurological symptoms |
| 28 | Early 40s | M | 4 days | Discectomy 25 years ago with severe pain. Intermittent low-level symptoms since | 111 | Yes: paramedics | No | No | Ambulance | MSK LBP, leg pain |
| 29 | Early 40s | F | 3 days | Previous LBP several episodes. MRI on 2 previous occasions. Specialist opinion abroad. NHS physio in UK | GP | Yes: GP | No | No | Lift | MSK LBP with neurological symptoms trauma: (fall) |
| 30 | Early 40s | M | Same day | Nil | 111 | Yes: GP | No | No | Lift | MSK LBP with neurological symptoms |
| 31 | Early 30s | M | Same day | 10-year history several acute episodes | 111 | Yes: 111 | No | Yes | Ambulance | MSK LBP with neurological symptoms trauma (lifting) |
| 32 | Early 70s | M | 1 day | Many year history low level symptoms | No | No | No | No | Drove self | MSK LBP likely vertebral fracture |
| 33 | Early 40s | M | Deteriorating over 4 weeks | Recurrent, severe symptoms; many years. This episode worst and longest | No | No | No | Yes | Lift | MSK radicular symptoms |
| 34 | Early 40s | M | 2 weeks radicular symptoms 24hrs sCES | V minor symptoms previously & no treatment | GP & HCP colleague | Yes: other | No | Yes | Lift | MSK LBP, radicular symptoms & CES symptoms |
| 35 | Early 50s | M | 3 days deteriorating back spasm making it difficult to breathe | Discectomy for sciatica 20 years previously. No ongoing symptoms but takes care to maintain activity levels and fitness or symptoms recur | GP E-consult; 111 | Yes: 999/111 | Yes | No | Ambulance/lift | MSK LBP then non-MSK LBP pneumonia |
| 36 | Late 20s | F | 2 weeks LBP, lump and 3 days of CES symptoms | Fall on coccyx several years before. No ongoing symptoms | GP E-consult | Yes: GP | Yes | No | Drove self/ lift | MSK LBP, radicular symptoms, sCES symptoms |
| 37 | Late 70s | M | 1 weeks severe LBP | Low level LBP | MIU, GP tel consult and paramedic assessment | No | No | No | Ambulance | ?MSK, investigations still in progress |
| 38 | Early 30s | M | 1 days | Low level occasionally | Attempted contact with GP: no appt for 2 weeks | No | No | No | Lift | MSK non-specific LBP |
| 39 | Mid 30s | M | 4-5 weeks prior to ED | Years on & off | Attempted contact with GP: no appt for 3 weeks | No | No | No | Lift | MSK non-specific LBP |
| 40 | Mid 60s | M | 3-4 weeks before ED | Nil | GP tel consult; Nurse and GP F2F. 111 (as deteriorated OOH). | Yes: 111 | No | No | Ambulance | Cause still unclear. A&E perceive this to be renal, patient MSK LBP |
| 41 | Mid 50s | F | 4 days low level symptoms agony that day | Nil | GP tel consults; 111; Community physio | Yes: GP 111 & physio | Yes | Yes | Ambulance/lift | Radicular symptoms |
| 42 | Late 40s | F | That day | One previous episode: 1 GP appt | Nil | No | No | No | Lift | MSK Non- specific LBP |
| 43 | Late 30s | M | 4 weeks worsening symptoms, worsening over 2 days before visit | Nil | GP tel consult | Suggested as an option by GP | No | No | Lift | LBP and radicular pain post trauma |
| 44 | Early 70s | M | 1 day before ED | 3 years progressively worsening LBP. One GP tel appt | Nil | No | No | No | Drove self | MSK non-specific LBP trauma (fall) |
| 45 | Late 30s | F | 1 day acute flare up | 18 months LBP | GP tel consult | Yes: GP | No | No | Taxi | MSK non-specific LBP |
| 46 | Mid 40s | M | That day first time | Several year LBP, intermittently moderately severe | 1^st^ time: 999 as first step second time GP post MRI abroad | Yes: GP | Yes | Not discussed | Lift | Radicular symptoms with sCES |
| 47 | Late 40s | F | 5 days | Nil | 111, GP, other | Yes: GP | No | No | Lift | Radicular symptoms, sCES |
