# Supplementary material for: Contrasting cultures of emergency department care: a qualitative study of patients’ experiences of attending the emergency department for low back pain in the UK
Source: BMJ Open. 2025 May 11;15(5):e091158. doi: 10.1136/bmjopen-2024-091158 (PMC12067803; doi:10.1136/bmjopen-2024-091158)
Supplement: Supplementary file 3 [file bmjopen-2024-091158.reviewer_comments.pdf]

PEER REVIEW HISTORY

BMJ Open publishes all reviews undertaken for accepted manuscripts. Reviewers are asked to complete a checklist review form and are provided with free text boxes to elaborate on their assessment. These free text comments are reproduced below.

ARTICLE DETAILS

Title (Provisional)

CONTRASTING CULTURES OF EMERGENCY DEPARTMENT CARE: A QUALITATIVE STUDY OF PATIENTS' EXPERIENCES OF ATTENDING THE EMERGENCY DEPARTMENT FOR LOW BACK PAIN IN THE UK

Authors

Ryan, Clare; Pope, Catherine J; Roberts, Lisa

VERSION 1 - REVIEW

**Reviewer**	**1**
**Name**	**Mescouto, Karime**
**Affiliation**	**The University of Queensland School of Health and Rehabilitation Sciences**
**Date**	**18-Nov-2024**
**COI**	**None**

Thank you for asking me to review "Attending the emergency department for low back pain: a qualitative study of patients' experiences". The authors present a very interesting and theoretically rich qualitative exploration of patients' experiences of seeking care for their LBP in EDs and offer that different ED cultures impact such experiences. Overall, I think that this manuscript could be an important addition to the literature. However, I feel that some major revisions need to be made, especially regarding rationale and methodological coherence. Please see my comments below:

Major Revisions

1. The entire rationale of the paper seems to centre on being "the first" to explore patients' experiences of attending ED for LBP. However, some papers are referenced in the introduction and discussion that investigated precisely that. My understanding is that the contribution of this paper is to provide a theoretically rich analysis of ED cultures that impact patients' experiences with ED. In addition, the use of a diverse population can also be considered a great addition. It would be beneficial to reflexively consider the paper's true contribution and explicitly mention how the present study converges/diverges from the

referenced studies. Consequently, the introduction and discussion would be more aligned with the rationale of the paper and explore the relevant literature in more depth.

2. Although I understand that the journal is mainly from a biomedical field, adding a theoretical underpinning section (even if brief) would be beneficial to guide the reader in understanding the findings and the narrative of the paper. Bourdieu's concepts are presented in the findings, which is slightly unusual. In addition, the paper could be more aligned with its theoretical underpinnings. If Bourdieu's concepts were used to guide the analysis, then it could be beneficial that the narrative of the introduction and discussion would also bring elements (within the area of LBP and ED) aligned with the concepts of field, habitus and professional identity theory. Further details on how and why the analysis moved from an inductive to a theory-driven approach would be beneficial.

Please see more detailed and minor revisions below:

Introduction

- The introduction would benefit from extensive revision to make the purpose of each paragraph in more targeted ways clearer to the reader and explain the reason the chosen references were being used. For example, the main purpose of the paragraph about ethnographic work seemed to be to provide a more in-depth understanding of what happens in ED services. The use of sociological concepts also helped to provide theoretically rich insights into exploring the services in different ways to explain key issues and barriers in ED departments. Instead of arguing that it is unknown how these concepts could be used in the context of LBP, it would be best to reflect on the main findings and how these are aligned or diverge from what has been done in the context of LBP (even if it compares to traditional quantitative studies – here it could be argued that qual studies that used sociological concepts were beneficial in providing insights into the sociocultural aspects of EDs that goes beyond individual's experiences to also attend to service's processes and workflow)s. Again, maybe a better link with the true contribution of this paper would guide what type of references are used in the introduction and the way they are used.
- Similarly, I would suggest making the rationale for presenting the references more robust than the argument that "the relevance of the findings to the UK setting is unknown". Perhaps exploring how references add to a more nuanced understanding of the ED experience and service and how the present study builds on these previous studies but provides a different and unique view would be more productive.
- There is an over-reliance on using parenthesis in sentence constructions that can be distracting. I would suggest a revision on such a need.

Methods:

- Would "designated and non-designated" mean "specialised and non-specialised" spinal centres?

- In the inclusion criteria, it seems that being able to communicate in English was not needed as there could be an option for interpreters. Is this correct? Please revise the wording.

- Is there a reason why including and not including physiotherapists in the staff was considered important to this study? I'm assuming that this was due to the research team's interest (as the main author is a physiotherapist?). Maybe briefly explaining the reason why would be beneficial.

- "[participants] were previously known to the researcher" Is this correct?

- "to provide the potential for credible, transferable findings" – I recommend reviewing the need for this justification regarding sample size as qualitative studies with 10 participants can also be credible and transferable. I'm assuming that a better explanation for the considerable number of participants would be to bring more diverse experiences from diverse participants and from multiple diverse EDs.

- "Interviews were continued until the maximum variation sample had been achieved and no significant new themes had arisen in two interviews". Does the concept of data saturation align with this paper's purpose and theoretical underpinnings? Maybe just use the concept of information power to justify the decided number of 50 participants? If this is a full interpretive qualitative study, that would be enough. Also, as this was a secondary analysis, the monthly meetings to discuss recruitment and sampling were part of the larger project, right? It would be beneficial to make this distinction clear.

Data analysis:

- It would be beneficial to explain the reasoning behind each analytical choice, as there seemed to be many. Why was Reflexive Thematic Analysis used? What does "ideal-type analysis" do? What is its main purpose, and how did the "types of ED experience" lead to the choice of this second analytical analysis? In addition, particular theoretical choices also guided the analysis, but these were not explored in the analysis section (only in the findings). I would suggest moving the theoretically driven work to a "theoretical underpinning" section and exploring a little more the interconnection between Bourdieu's concepts and identity theory. I'm assuming there was an intention to combine micro, meso, and macro-level processes to explain peoples' diverse experiences in ED and ED's diverse cultures. This (or perhaps another reason) could be clearer.

Findings:

- There is not as much diversity in the sample as one would have expected after reading the description of the data collection strategy. The main diversity seems to be from the participants' socioeconomic profiles. Also, from those outside of the UK, what does being in the deciles according to the Index of Multiple Deprivation mean? This could be added or simply mentioned in other words when describing the participants.

- To be aligned with this study's theoretical underpinnings and analytical tools, I would suggest changing the wording "We found", as the researchers interpreted and constructed the findings.

- The year on Bourdieu's reference is incorrect. Also, this reference does not appear in the reference list.

- What would be "ED majors and minors"?

- The findings are very interesting and insightful. Some small suggestions:

Would there be a more representative title for the first culture? It seems like all the cultures encompassed emergency screening (as expected), but these screenings were done and performed differently – yielding different patients' experiences. Would something within the lines of "cold biomedical agility" better represent this first culture? There are always underlying reasons why people attend EDs rather than a proper medical urgency (e.g., fear, lack of community access, need for medication, etc), but that should also be acknowledged and addressed somehow (as the third culture in the findings suggests).

Review the need to add "labelled using an in-vivo code"

In "a culture of kindness" there are references when describing the findings. Although I know this is common in sociology, because the other "cultures" did not present the findings in the same way, I suggest being consistent with the presentation within a more traditional/biomedically focused journal or having a similar presentation on the other parts of the finding.

Within this same section, it becomes clear why there was a focus on understanding the physiotherapist's role in ED (besides the main author being a physiotherapist). I suggest adding some of this justification in the methods section.

Would it be only "kindness" or more of "appropriate and kind care"? Although I completely understand the intent behind the word "kindness", sometimes it is used with the underlying assumption that accessing ethical and dignified care is a luxury and a favour that healthcare providers do for patients rather than their job and a human right.

Discussion

- It would be beneficial to explore the context of participants' demographics to argue for more appropriate and kind care (not only related to a biopsychosocial approach). Considering that most of the participants were from a low SES, it would be important to discuss their usual difficulty with accessing healthcare and their likelihood of being perceived with suspicion, cynicism, and stigma by healthcare professionals. Although it was mentioned that the key strength was to enable the voices of underserved populations, this was not highlighted in the discussion.

- There have been studies that explored the role of physiotherapists in EDs that could be added. Please see some examples below:

Chrobok L, Espejo T, Riedel HB, Kirchberger J, Overberg JA, Felber F, Perrot G, Nickel CH, Bingisser R. On-Site Physiotherapy in Emergency Department Patients Presenting with Nonspecific Low Back Pain: A Randomized Controlled Trial. J Clin Med. 2024 May 27;13(11):3149. doi: 10.3390/jcm13113149. PMID: 38892860; PMCID: PMC11173222.

Matifat E, Berger Pelletier E, Brison R, Hébert LJ, Roy JS, Woodhouse L, Berthelot S, Daoust R, Sirois MJ, Booth R, Gagnon R, Miller J, Tousignant-Laflamme Y, Emond M, Perreault K, Desmeules F. Advanced practice physiotherapy care in emergency departments for patients with musculoskeletal disorders: a pragmatic cluster randomised controlled trial and cost analysis. Trials. 2023 Feb 6;24(1):84. doi: 10.1186/s13063-023-07100-x.

**Reviewer**	**2**
**Name**	**Truter, Piers**
**Affiliation**	**The University of Notre Dame Australia, School of Health Sciences**
**Date**	**18-Nov-2024**
**COI**	**None**

This has been an enjoyable paper to review on an important topic. Low Back Pain is highly prevalent in ED, patients have diverse clinical needs and ED staff struggle with this presentation type due to their disabling pain. Speaking as a long standing ED clinician, the three identified cultures have a ring of authenticity even in EDs in another country. This paper will make an important contribution to EDs reviewing their professional cultures as it pertains to providing high quality care.

Review

Page numbers from original numbering not whole submission numbering.

Overview:

This has been an enjoyable paper to review on an important topic. Low Back Pain is highly prevalent in ED, patients have diverse clinical needs and ED staff struggle with this presentation type due to their disabling pain. Speaking as a long standing ED clinician, the three identified cultures have a ring of authenticity even in EDs in another country. This paper will make an important contribution to EDs reviewing their professional cultures as it pertains to providing high quality care.

While there are many points below, there are three main issues for your consideration;

1) There is a critical issue with the population included in this paper. Please consider including comprehensive inclusion / exclusion criteria and justification for these. The mingling of MSK and non-MSK LBP is potentially confounding.

Patients with non-MSK LBP may have very different presentations, requirements for assessment / treatment and significantly different follow up. There may be differences in clinical staff approaches to LBP vs gastro vs gynae problems.

2) Consider providing a 'setting' section in the methods that paints a picture of the ED environment as it is operationalised in the UK. This will improve understanding and relatability for international readers. For instance, the local ED here in Perth Australia is attached to a 750 bed tertiary hospital and caters to 350+ patients a day. There are separate entries for adults and children (there is a dedicated Childen's ED). The ED has around 90 beds. There is a 20 bed 'short stay ward' attached to the ED.
3) Consider review of the discussion. The strongest finding is about culture of EDs. This should be the first point addressed in the discussion. How can this be influenced. Consider focusing on the voice from the patients in the 'implications'. There is a clear call to action on access, follow up and completeness of care. Also consider whether the next step is establishing an 'ED LBP guideline' or if it is addressing identified ED cultures that are at odds with the contents of the many excellent LBP guidelines.

Abstract – consider review in light of any changes to discussion / implications

Page 3 Line 48 – an important part of clinical guidelines is conducting a differential diagnostic process to exclude sinister causes that mimic LBP. It is only after these have been excluded that a BSP approach and supported self-management are advised.

Page 4 Line 15 – Consider also that it is not just the severity of pain, but also how the person experiencing the pain interprets this signal as a sign of a critical health issue (not a condition that can be safely managed in the community)

Page 4 Line 31 – please clarify the nature and context of 'tensions'. i.e. are these tensions patient or system?

Page 5 Line 5 – This is a reasonable point, although Graham etal. Includes articles from the USA, which does not have a publicly funded health system. Possibly consider …countries with **mostly** publicly funded….

Page 6 Line 55 – "…and were previously known to the researcher." Please clarify exactly what this means and the relationship between the participants and the researcher (is this CR?).

Page 6 Line 52 – It is not clear what population of people attending ED are included in this paper or how they were selected. Was this group selected from their presenting complaint (i.e. perspective on their condition prior to ED treatment) or from their ED

diagnosis? This is a critical issue for the paper, as reading on to Supplementary Material Two, there is a mixed group of MSK LBP and other non-MSK issues (e.g. gastro / gynae issues). The care requirements for MSK LBP and medical issues are not the same.

Page 10 – Figure 1 is not complete, there is text that is not legible at the bottom of the box.

Page 11 Lines 52-57 – it is not clear what point you are making here. Please clarify.

Page 12 Line 33 - …the decision to attend. – this appears to be an incomplete sentence.

Page 12 Line 37 – what is the ED offer?

General Comment: ED Majors and Minors are terms with local meaning. Please clarify these terms early in the paper for an international reader. Are their differences in access / staffing / treatment options? How to people end up at one or the other? Maybe add a section in Methods to give a more complete view of the ED settings.

Page 17 Line 13 - …identified significant variation in *the patient experience* of ED care for LPB.

Page 17 – general comment – the major finding of this paper is that there is a patient perception of three ED professional cultures and that these cultures define access and quality of care in the ED. It would suggest that the first call to action would be to explore ways to address the ED cultural issues – with a specific focus on understanding the needs of patients who have taken the time to present to the ED (nobody does this without purpose).

Page 17 – 2nd general comment – is the issue a lack of guidelines for ED care for low back pain? Or is it a lack of adoption of these and potentially a cultural / pragmatic clash with the BSP components?

Page 18 general comment – the other major issue appears to be a lack of access to an effective pathway of care. This includes entering a pathway of care (receiving an urgent assessment and treatment that meets patient needs) and then progressing down that pathway with accessible and well signposted elements of follow up (medication, plan, next clinical review).

Should you wish to discuss or clarify, please feel free to contact
(piers.truter@nd.edu.au)

**Reviewer**	**3**
**Name**	**Kim, Howard**

Affiliation **Northwestern University Feinberg School of Medicine, Department of Emergency Medicine**

Date **20-Nov-2024**

COI **None**

This is a well-conducted and well-written qualitative study. It confirms the findings of a couple other similar papers from other countries/contexts, and this study has the added advantage of being from multiple EDs. I have some suggestions and comments for improvement:

1. Primacy. The authors state multiple times that this is the first qualitative study of ED patient experiences re: low back pain (Page 8, Line 33; Page 18, Line 50, etc…), yet they reference a few other qualitative studies that have examined this exact question, so this isn't an accurate statement. I don't think you need to establish primacy for this study to be impactful. Its findings can be important confirmation (using superior sampling/methods) of those prior studies.

2. Bordeau's concepts of field and theory. I find the frequent weaving of Bordeau's concepts of field and theory into the Results section to be somewhat distracting and casting some doubt on whether the qualitative analysis was truly inductive. I think it would be best to remove these references from the Results section (as the paragraphs dedicated to Bordeau's fields in each of the 3 themes do not actually present study data); they could be moved to the Discussion. Alternatively, if you want to retain this text in Results, I think you need to more formally describe Bordeau's theories in Methods and how you used these theories to inform the content analysis – I think it's ok to use some pre-existing theory to inform coding, but I think you need to clearly state this in advance.

3. Page 7, Line 49: It would be helpful for non-UK readers if you gave additional context on how frequently physiotherapists work in the ED and what role they serve (e.g., extended scope, independent practitioner, etc…). U.S. readers are not familiar with the idea of PTs serving as independent practitioners in the ED as we are a bit backwards and only have PTs in a secondary/consulting role.

4. Page 8, Line 33: Please clarify when interviews were conducted relative to the index ED visit. Please also specify the dates from which index ED visits were drawn (you specify only the dates for when qualitative interviews were conducted).

5. Page 9, Line 8: What were the other two languages spoken?

6. Page 9, Line 35: You describe a first/parent paper a couple times in this manuscript (also on Page 10, Line 10). It would be helpful to the reader to know more about that parent study (i.e., its objective, eligibility criteria, etc…) if this study is considered a secondary analysis. The objective and design of that parent study could affect the way in which participants were enrolled and data were collected for this study.

7. Figure 1: This figure was cut off and there appears to be some text that is missing. I would recommend putting these data in Table format, as this is an unusual presentation of demographic characteristics and does not have added value as a Figure.

8. Figure 1: What were the number of participants from each site?

9. Figure 1: Were these data ascertained from the electronic medical record, interviews, or some other source? Some of the data (LBP presentation, symptom duration, LBP history) require some subjective interpretation and so it would be important to specify how data were collected and who extracted these data and how.

10. Figure 1, LBP presentation. 14 of 47 participants (30%) having symptoms suggestive of cauda equinae is extraordinarily high. This is much higher than any study of ED LBP that I have ever read and much higher than I have observed in clinical practice. I think you need to specify what the operational definition of “symptoms suggest of cauda equinae” was for this study. I might expect 30% of participants to report some subjective numbness/tingling in the back/buttocks/leg/foot, but this is not a cauda equinae symptom (unless it is saddle anesthesia).

11. Page 12, Line 48: You mentioned that Themes 2 and 3 were also in vivo codes, but you did not mention that here. Was this also an in vivo code?

12. Page 13, Line 32: I’m not familiar with how this term (signposted) is being used here – do you mean referred?

13. Page 14, Line 7: You mention majors and minors a few times in this manuscript (also on Page 16, Line 18); please define these terms. It sounds like this refers to the triage acuity of the ED visit and the subsequent zoning of patients into a particular part of the ED – I suggest including parenthetical synonyms (e.g., low acuity, “fast track”) to facilitate readership.

14. Page 14, Line 9: You say doctors or nurses here (as opposed to physiotherapists), but I think you might mean “nurse practitioners” as it appears that nurses evaluate patients regardless of whether a physician vs physiotherapist is the primary ED provider.

15. Page 15, Line 25: Do you have additional quotes to substantiate this “gatekeeper” code/theme? You use the term “gatekeeping” many times throughout the manuscript (and even in the prior Theme 1). I would like to see some other quotes that reinforce this as a predominant theme given how frequently you refer to it. We usually think of “gatekeeping” in terms of restricting access to advanced imaging (MRI) or hospital admission, but this quote is a little unusual because the MRI has already been obtained so it’s not clear what is being gatekept here (i.e., the ED has little else to offer beyond this).

VERSION 1 - AUTHOR RESPONSE

Reviewer	Point no.	Point to be addressed	How point made is addressed	Where the amendment has been made
Reviewer 1 Dr. Karime Mescouto	1.	The entire rationale of the paper seems to centre on being "the first" to explore patients' experiences of attending ED for LBP. However, some papers are referenced in the introduction and discussion that investigated precisely that. My understanding is that the contribution of this paper is to provide a theoretically rich analysis of ED cultures that impact patients' experiences with ED. In addition, the use of a diverse population can also be considered a great addition. It would be beneficial to reflexively consider the paper's true contribution and explicitly mention how the present study converges/diverges from the referenced studies. Consequently, the introduction and discussion would be more aligned with the rationale of the paper and explore the relevant literature in more depth.	The justification for the paper has been revised as suggested to provide a theoretically rich analysis of ED cultures that impact patients' experiences with ED.<br><br>The introduction has been redrafted to more clearly articulate the salience of each of the included studies.	Abstract p2, summary of results section p12, conclusion 20.
	2.	Although I understand that the journal is mainly from a biomedical field, adding a theoretical underpinning section	The section summarising the theory employed has been moved to the methods section.	Methods: Data analysis, p8-9

		(even if brief) would be beneficial to guide the reader in understanding the findings and the narrative of the paper. Bourdieu's concepts are presented in the findings, which is slightly unusual.<br>In addition, the paper could be more aligned with its theoretical underpinnings.<br><br>If Bourdieu's concepts were used to guide the analysis, then it could be beneficial that the narrative of the introduction and discussion would also bring elements (within the area of LBP and ED) aligned with the concepts of field, habitus and professional identity theory.<br><br>Further details on how and why the analysis moved from an inductive to a theory-driven approach would be beneficial.	I have not referred in the literature review to studies that have employed Bourdieu's theory or ED culture as this literature was not consulted prior to undertaking the analysis.<br><br>These details have been clarified	
	3. Introduction	The introduction would benefit from extensive revision to make the purpose of each paragraph in more targeted ways clearer to the reader and explain the reason the chosen references were being used. For example, the main purpose of the paragraph about ethnographic work seemed to be to	Thank you for these suggestions. The introduction has been revised to better articulate the salience of each of the included literatures and to highlight how together the findings suggest a disparity between patients' priorities in ED care and concepts that inform how ED care is provided.<br><br>I have deliberately limited the literature included to qualitative research to convey	Introduction p3-5

		provide a more in-depth understanding of what happens in ED services. The use of sociological concepts also helped to provide theoretically rich insights into exploring the services in different ways to explain key issues and barriers in ED departments. Instead of arguing that it is unknown how these concepts could be used in the context of LBP, it would be best to reflect on the main findings and how these are aligned or diverge from what has been done in the context of LBP (even if it compares to traditional quantitative studies – here it could be argued that qual studies that used sociological concepts were beneficial in providing insights into the sociocultural aspects of EDs that goes beyond individual's experiences to also attend to service's processes and workflows). Again, maybe a better link with the true contribution of this paper would guide what type of references are used in the introduction and the way they are used. Similarly, I would suggest making the	the rich insights of these studies and to keep the background to a reasonable length.<br><br>I have not referred in the literature review to studies that have employed Bourdieu's theory or ED culture as this literature was not consulted prior to undertaking the analysis.	

		rationale for presenting the references more robust than the argument that "the relevance of the findings to the UK setting is unknown". Perhaps exploring how references add to a more nuanced understanding of the ED experience and service and how the present study builds on these previous studies but provides a different and unique view would be more productive.		
	4.	There is an over-reliance on using parenthesis in sentence constructions that can be distracting. I would suggest a revision on such a need.	Revised to remove parentheses where appropriate.	Throughout the paper
	5. Methods:	Would "designated and non-designated" mean "specialised and non-specialised" spinal centres?	Wording has been amended to 'regional spinal centres'.	Methods: Setting, participants and recruitment, P6
	6.	In the inclusion criteria, it seems that being able to communicate in English was not needed as there could be an option for interpreters. Is this correct? Please revise the wording.	The criterion of being able to communicate in English has been removed.	Methods Setting, participants and recruitment, , p6
	7.	Is there a reason why including and not including physiotherapists in the staff was considered important	The reason for including the literature about how it is to be managed in the ED by a physiotherapists and the relevance of including recruiting sites who employed	Introduction p4 and Setting, participants and recruitment P6

		to this study? I'm assuming that this was due to the research team's interest (as the main author is a physiotherapist?). Maybe briefly explaining the reason why would be beneficial.	physiotherapists in the staff skill mix has been added in the introduction and methods sections respectively.	
	8.	"[participants] were previously known to the researcher" Is this correct?	This was a typo and has been amended to "…and were not previously known to the researcher."	Methods: Setting, participants and recruitment P6
	9.	"to provide the potential for credible, transferable findings" – I recommend reviewing the need for this justification regarding sample size as qualitative studies with 10 participants can also be credible and transferable. I'm assuming that a better explanation for the considerable number of participants would be to bring more diverse experiences from diverse participants and from multiple diverse EDs.	The text has been amended to 'We aimed to recruit up to 50 participants, a number considered appropriate to enable in-depth inquiry and to align with our maximum variation sampling strategy'.	Methods: Setting, participants and recruitment P7
	10.	"Interviews were continued until the maximum variation sample had been achieved and no significant new themes had arisen in two interviews". Does the concept of data saturation align with this paper's purpose and theoretical underpinnings? Maybe just use the concept of	Amended as suggested. This section now reads: 'Interviews were continued until the maximum variation sample had been achieved, with the 47 interviews providing the breadth and depth of data sought.'<br><br>The decision to explore patients' experiences of ED care for LBP was made a	Methods: Data collection P7 penultimate paragraph

		information power to justify the decided number of 50 participants? If this is a full interpretive qualitative study, that would be enough.<br><br>Also, as this was a secondary analysis, the monthly meetings to discuss recruitment and sampling were part of the larger project, right? It would be beneficial to make this distinction clear.	priori and therefore decisions about recruitment and sampling related in part to the data explored in this study.	
	11. Data analysis	It would be beneficial to explain the reasoning behind each analytical choice, as there seemed to be many. Why was Reflexive Thematic Analysis used? What does “ideal-type analysis” do? What is its main purpose, and how did the “types of ED experience” lead to the choice of this second analytical analysis?<br><br>In addition, particular theoretical choices also guided the analysis, but these were not explored in the analysis section (only in the findings). I would suggest moving the theoretically driven work to a “theoretical underpinning” section and exploring a little more the	These details have been clarified.<br><br>The section summarising the theory employed has been moved to the methods section.<br><br>Rather than exploring micro, meso and macro processes, our analysis sought to explore the characteristics of the different ED cultures that were important to patients and to draw on theory to help us make sense of our findings.	Methods: Data analysis p8-9<br><br>Methods: Data analysis, p8-9

		interconnection between Bourdieu's concepts and identity theory.<br><br>I'm assuming there was an intention to combine micro, meso, and macro-level processes to explain peoples' diverse experiences in ED and ED's diverse cultures. This (or perhaps another reason) could be clearer.		
	12. Findings	There is not as much diversity in the sample as one would have expected after reading the description of the data collection strategy. The main diversity seems to be from the participants' socioeconomic profiles. Also, from those outside of the UK, what does being in the deciles according to the Index of Multiple Deprivation mean? This could be added or simply mentioned in other words when describing the participants.<br>To be aligned with this study's theoretical underpinnings and analytical tools, I would suggest changing the wording "We found", as the researchers interpreted and constructed the findings.	I have now highlighted the ways in which underserved populations were included in the sample.	Findings: Setting and sample characteristics<br>p10

	13.	The year on Bourdieu's reference is incorrect. Also, this reference does not appear in the reference list.	The intext citation has been amended and the reference added to the reference list.	Throughout text and reference list
	14.	What would be "ED majors and minors"?	These terms have been defined in the methods section and in the text as high and low acuity treatment areas within the ED.	Findings: Setting and sample characteristics p9
	15.	Would there be a more representative title for the first culture? It seems like all the cultures encompassed emergency screening (as expected), but these screenings were done and performed differently – yielding different patients' experiences. Would something within the lines of "cold biomedical agility" better represent this first culture? There are always underlying reasons why people attend EDs rather than a proper medical urgency (e.g., fear, lack of community access, need for medication, etc), but that should also be acknowledged and addressed somehow (as the third culture in the findings suggests).	Thank you for the suggestion. I have retained the use of a culture of emergency screening only as participants did not refer to staff manner when describing this culture of ED care.	Findings: a culture of emergency screening only, p12
	16.	Review the need to add "labelled using an in-vivo code"<br>In "a culture of kindness" there are references when describing the findings. Although I know this is common	The title for this culture has been amended, as suggested to a culture of appropriate and kind care.	Findings: a culture of appropriate and kind care p16

		in sociology, because the other "cultures" did not present the findings in the same way, I suggest being consistent with the presentation within a more traditional/biomedically focused journal or having a similar presentation on the other parts of the finding.		
	17.	Within this same section, it becomes clear why there was a focus on understanding the physiotherapist's role in ED (besides the main author being a physiotherapist). I suggest adding some of this justification in the methods section.	Justification now included.	Setting, participants and recruitment P6 first paragraph
	18.	Would it be only "kindness" or more of "appropriate and kind care"? Although I completely understand the intent behind the word "kindness", sometimes it is used with the underlying assumption that accessing ethical and dignified care is a luxury and a favour that healthcare providers do for patients rather than their job and a human right.	Thank you, we agree, and a culture of appropriate and kind care has been used.	Findings P16
	19. Discussion	It would be beneficial to explore the context of participants' demographics to argue for more	Thank you, this important issue has now been picked up in the analysis and discussion.	Analysis p16 final paragraph Discussion, p19

		appropriate and kind care (not only related to a biopsychosocial approach). Considering that most of the participants were from a low SES, it would be important to discuss their usual difficulty with accessing healthcare and their likelihood of being perceived with suspicion, cynicism, and stigma by healthcare professionals. Although it was mentioned that the key strength was to enable the voices of underserved populations, this was not highlighted in the discussion.		
	20.	There have been studies that explored the role of physiotherapists in EDs that could be added. Please see some examples below: Chrobok L, Espejo T, Riedel HB, Kirchberger J, Overberg JA, Felber F, Perrot G, Nickel CH, Bingisser R. On-Site Physiotherapy in Emergency Department Patients Presenting with Nonspecific Low Back Pain: A Randomized Controlled Trial. J Clin Med. 2024 May 27;13(11):3149. doi: 10.3390/jcm13113149. PMID: 38892860; PMCID: PMC11173222.	Thank you. Whilst there are a number of primary studies and systematic reviews that quantitatively explore the clinical and cost effectiveness of physiotherapists being integrated into the ED skill mix, to align with the research question and the journal word count, we elected to include only studies that qualitatively explored patients' experiences of being managed by a physiotherapist in the ED.	Introduction

		Matifat E, Berger Pelletier E, Brison R, Hébert LJ, Roy JS, Woodhouse L, Berthelot S, Daoust R, Sirois MJ, Booth R, Gagnon R, Miller J, Tousignant-Laflamme Y, Emond M, Perreault K, Desmeules F. Advanced practice physiotherapy care in emergency departments for patients with musculoskeletal disorders: a pragmatic cluster randomised controlled trial and cost analysis. Trials. 2023 Feb 6;24(1):84. doi: 10.1186/s13063-023-07100-x.		
Reviewer 2 Dr Piers Truter	Major points 1.	There is a critical issue with the population included in this paper. Please consider including comprehensive inclusion / exclusion criteria and justification for these. The mingling of MSK and non-MSK LBP is potentially confounding. Patients with non-MSK LBP may have very different presentations, requirements for assessment / treatment and significantly different follow up. There may be differences in clinical staff approaches to LBP vs gastro vs gynae problems.	The decision to include people with all types of LBP, including non-MSK causes was deliberate. This relates in part to the inclusion criteria of the primary study which explored why people attend the ED for LBP. This population often will not be able to distinguish between MSK and non-MSK causes. Furthermore, this study aimed to explore patients' experiences of ED care for all types of LBP. This rationale has been clarified in the text.	Methods: Setting, participants and recruitment p6

	2.	Consider providing a 'setting' section in the methods that paints a picture of the ED environment as it is operationalised in the UK. This will improve understanding and relatability for international readers. For instance, the local ED here in Perth Australia is attached to a 750 bed tertiary hospital and caters to 350+ patients a day. There are separate entries for adults and children (there is a dedicated Childen's ED). The ED has around 90 beds. There is a 20 bed 'short stay ward' attached to the ED.	A setting section has been included as suggested.	Methods: Setting, participants and recruitment p6
	3.	Consider review of the discussion. The strongest finding is about culture of EDs. This should be the first point addressed in the discussion. How can this be influenced. Consider focusing on the voice from the patients in the 'implications'. There is a clear call to action on access, follow up and completeness of care. Also consider whether the next step is establishing an 'ED LBP guideline' or if it is addressing identified ED cultures that are at odds with the contents of the many excellent LBP guidelines.	The discussion has been revised to address these issues	Discussion p17-19

	Additional points 4.	Abstract – consider review in light of any changes to discussion / implications	Abstract amended	Abstract p2
	5.	Page 3 Line 48 – an important part of clinical guidelines is conducting a differential diagnostic process to exclude sinister causes that mimic LBP. It is only after these have been excluded that a BSP approach and supported self-management are advised.	Amended to include the words ‘following screening to exclude serious pathology’.	Introduction paragraph 1 p3
	6.	Page 4 Line 15 – Consider also that it is not just the severity of pain, but also how the person experiencing the pain interprets this signal as a sign of a critical health issue (not a condition that can be safely managed in the community)	This sentence has been amended to reflect this.	Introduction p4 paragraph 1
	7.	Page 4 Line 31 – please clarify the nature and context of ‘tensions’. i.e. are these tensions patient or system?	This sentence has been amended.	Introduction p4 paragraph 2
	8.	Page 5 Line 5 – This is a reasonable point, although Graham etal. Includes articles from the USA, which does not have a publicly funded health system. Possibly consider …countries with mostly publicly funded….	This sentence has been removed to reduce the word count.	Introduction p4 paragraph 3

	9.	Page 6 Line 55 – "…and were previously known to the researcher." Please clarify exactly what this means and the relationship between the participants and the researcher (is this CR?).	This was a typo and has been amended to "…and were not previously known to the researcher."	Methods: Setting, participants and recruitment p6
	10.	Page 6 Line 52 – It is not clear what population of people attending ED are included in this paper or how they were selected. Was this group selected from their presenting complaint (i.e. perspective on their condition prior to ED treatment) or from their ED diagnosis? This is a critical issue for the paper, as reading on to Supplementary Material Two, there is a mixed group of MSK LBP and other non-MSK issues (e.g. gastro / gynae issues). The care requirements for MSK LBP and medical issues are not the same.	Adults with all types of LBP were included. The only exclusion criteria were people who did not have the capacity to consent, and people known to the researcher. As detailed in point one above, this study aimed to explore the experiences of patients who attended for all types of LBP.	Methods: Setting, participants and recruitment paragraph 2, p6
	11.	Page 10 – Figure 1 is not complete, there is text that is not legible at the bottom of the box.	Figure 1 line boundaries amended so that text is legible.	Findings: Box1 p11
	12.	Page 11 Lines 52-57 – it is not clear what point you are making here. Please clarify.	Amended	Findings: p12
	13.	Page 12 Line 33 - …the decision to attend. –	Amended	Findings: p13

		this appears to be an incomplete sentence.		
	14.	Page 12 Line 37 – what is the ED offer? General Comment: ED Majors and Minors are terms with local meaning. Please clarify these terms early in the paper for an international reader. Are their differences in access / staffing / treatment options? How to people end up at one or the other? Maybe add a section in Methods to give a more complete view of the ED settings.	The terms majors and minors have been defined in the findings section and in the text in parentheses as high and low acuity treatment areas within the ED.	Findings: Setting and sample p9
	15.	Page 17 Line 13 - …identified significant variation in the patient experience of ED care for LPB.	Amended.	Findings p18
	16.	Page 17 – general comment – the major finding of this paper is that there is a patient perception of three ED professional cultures and that these cultures define access and quality of care in the ED. It would suggest that the first call to action would be to explore ways to address the ED cultural issues – with a specific focus on understanding the needs of patients who have taken the time to present to the ED (nobody does this without purpose).	Thank you. The discussion has been redrafted to reflect these points.	Discussion p17-19

	17.	Page 17 – 2nd general comment – is the issue a lack of guidelines for ED care for low back pain? Or is it a lack of adoption of these and potentially a cultural / pragmatic clash with the BSP components?	We agree, the discussion has been amended to reflect this.	Discussion p17-19
	18.	Page 18 general comment – the other major issue appears to be a lack of access to an effective pathway of care. This includes entering a pathway of care (receiving an urgent assessment and treatment that meets patient needs) and then progressing down that pathway with accessible and well signposted elements of follow up (medication, plan, next clinical review).	The discussion has been amended to include this	Discussion p18 and p19
Reviewer 3 Dr. Howard Kim	1.	Primacy. The authors state multiple times that this is the first qualitative study of ED patient experiences re: low back pain (Page 8, Line 33; Page 18, Line 50, etc…), yet they reference a few other qualitative studies that have examined this exact question, so this isn't an accurate statement. I don't think you need to establish primacy for this study to be impactful. Its findings can be important confirmation (using superior	The justification for the paper has been revised as suggested to provide a theoretically rich analysis of ED cultures that impact patients' experiences with ED.	Abstract p2, summary of results section p12, conclusion p19/20.

		sampling/methods) of those prior studies.		
	2.	Bordeau's concepts of field and theory. I find the frequent weaving of Bordeau's concepts of field and theory into the Results section to be somewhat distracting and casting some doubt on whether the qualitative analysis was truly inductive. I think it would be best to remove these references from the Results section (as the paragraphs dedicated to Bordeau's fields in each of the 3 themes do not actually present study data); they could be moved to the Discussion. Alternatively, if you want to retain this text in Results, I think you need to more formally describe Bordeau's theories in Methods and how you used these theories to inform the content analysis – I think it's ok to use some pre-existing theory to inform coding, but I think you need to clearly state this in advance.	The analysis included both inductive and deductive phases. The theoretically informed analysis occurred in response to the findings of the deductive analysis and to help explain these findings. I have moved the description of Bourdieu's theories of field and habitus in the methods: data analysis section.	Methods: data analysis p8-9
	3.	Page 7, Line 49: It would be helpful for non-UK readers if you gave additional context on how frequently physiotherapists work in the ED and what role they serve (e.g.,	Brief details added re this to the methods section justifying the variation sought in recruiting sites.	Findings: Setting and sample characteristics p9

		extended scope, independent practitioner, etc…). U.S. readers are not familiar with the idea of PTs serving as independent practitioners in the ED as we are a bit backwards and only have PTs in a secondary/consulting role.		
	4.	Page 8, Line 33: Please clarify when interviews were conducted relative to the index ED visit. Please also specify the dates from which index ED visits were drawn (you specify only the dates for when qualitative interviews were conducted).	This data was not collected. Contact was however made with potential participants within several days of receiving their contact details. In almost all cases this was within a week of attending the ED.	
	5.	Page 9, Line 8: What were the other two languages spoken?	This information has not been retained.	
	6.	Page 9, Line 35: You describe a first/parent paper a couple times in this manuscript (also on Page 10, Line 10). It would be helpful to the reader to know more about that parent study (i.e., its objective, eligibility criteria, etc…) if this study is considered a secondary analysis. The objective and design of that parent study could affect the way in which participants were enrolled and data were collected for this study.	Details of the multisite study and the other paper that draws on this dataset is now described in the method.	Method: study design p5

	7.	Figure 1: This figure was cut off and there appears to be some text that is missing. I would recommend putting these data in Table format, as this is an unusual presentation of demographic characteristics and does not have added value as a Figure.	Figure amended but retained as a figure.	Findings: Box1 p11
	8.	Figure 1: What were the number of participants from each site?	Numbers of participants recruited from each site added to the method.	Findings: setting and sample characteristics , p9
	9.	Figure 1: Were these data ascertained from the electronic medical record, interviews, or some other source? Some of the data (LBP presentation, symptom duration, LBP history) require some subjective interpretation and so it would be important to specify how data were collected and who extracted these data and how.	As detailed in the method, patients' sociodemographic characteristics were collected at the end of the interview. As detailed in Figure, the type of LBP was based on CR's interpretation of the information discussed during the interview.	Method: setting, participants and recruitment p6
	10.	Figure 1, LBP presentation. 14 of 47 participants (30%) having symptoms suggestive of cauda equinae is extraordinarily high. This is much higher than any study of ED LBP that I have ever read and much higher than I have observed in clinical practice. I think you need to specify what the operational definition	Thank you. Purposive sampling is not intended to be representative of the population who attend the ED. We did limit the numbers recruited with sCES, recognising that the proportion of the sample with sCES was high. Our definition of symptoms consistent with suspected CES aligns with UK GIRFT national suspected CES pathway guidance (2023). https://gettingitrightfirsttime.co.uk/wp-	Findings: Box 1 p11

		of "symptoms suggest of cauda equinae" was for this study. I might expect 30% of participants to report some subjective numbness/tingling in the back/buttocks/leg/foot, but this is not a cauda equinae symptom (unless it is saddle anesthesia).	content/uploads/2024/07/National-Suspected-Cauda-Equina-Pathway-Updated-July-2024.pdf.	
	11	Page 12, Line 48: You mentioned that Themes 2 and 3 were also in vivo codes, but you did not mention that here. Was this also an in vivo code?	Theme one 'emergency screening only' was not an in vivo code.	Findings: A culture of emergency screening only p12
	12	Page 13, Line 32: I'm not familiar with how this term (signposted) is being used here – do you mean referred?	Text amended to ' and to refer on or advise patients..'	Findings: p13 paragraph starting 'we argue...'
	13	Page 14, Line 7: You mention majors and minors a few times in this manuscript (also on Page 16, Line 18); please define these terms. It sounds like this refers to the triage acuity of the ED visit and the subsequent zoning of patients into a particular part of the ED – I suggest including parenthetical synonyms (e.g., low acuity, "fast track") to facilitate readership.	The terms majors and minors have been defined in the findings section and in the text in parentheses as high and low acuity treatment areas within the ED.	Findings: Setting and sample, first paragraph p9.
	14	Page 14, Line 9: You say doctors or nurses here (as opposed to physiotherapists), but I think you might	In the UK, Physiotherapists can assess and manage patients in the ED autonomously. In contrast, nurses (including Advanced	Findings: Setting and sample, first paragraph, p9.

		mean “nurse practitioners” as it appears that nurses evaluate patients regardless of whether a physician vs physiotherapist is the primary ED provider.	Nurse Practitioners) are currently required to discuss their management of patients with a medic. This has been clarified in the paper.	
	15	Page 15, Line 25: Do you have additional quotes to substantiate this “gatekeeper” code/theme? You use the term “gatekeeping” many times throughout the manuscript (and even in the prior Theme 1). I would like to see some other quotes that reinforce this as a predominant theme given how frequently you refer to it. We usually think of “gatekeeping” in terms of restricting access to advanced imaging (MRI) or hospital admission, but this quote is a little unusual because the MRI has already been obtained so it’s not clear what is being gatekept here (i.e., the ED has little else to offer beyond this).	Thank you. In theme one of the findings, I have clarified that examples of the resources that staff in this culture elected not to use included staff time to validate patients’ symptoms, undertake a thorough biopsychosocial assessment, to discuss the diagnosis, prognosis and optimal management or to refer or signpost (advise) patients as to how to access appropriate follow up care. This gatekeeping of ED resources is evident in the data extracts included.	Findings: A culture of emergency screening only, paragraph starting ‘we argue…’ p13

VERSION 2 - REVIEW

**Reviewer**	**2**
**Name**	**Truter, Piers**

Affiliation **The University of Notre Dame Australia, School of Health Sciences**

Date **20-Feb-2025**

COI

Thank you for the hard work put in to revise this manuscript. It is reading well.

There is only one small issue describe below about clarity on how the participants were selected with reference to their presenting complaint and final ED diagnosis.

COMMENTS

Is there a typo in the first methods paragraph on study design? Should the '3' be there?

“Ethical approval was gained from the West of Scotland Research Ethics Committee3 in June 2021 (ref 21/WS/0068).”

This is an argument from the introduction;

“This literature argues that key relevant concepts include the primacy of the lifesaving function of the ED; gatekeeping of ED resources (to align with the ED’s primary function); the moral evaluation of patients perceived by staff to have attended illegitimately (those who do not require this acuity of care); and reasonableness (circumstances that make the attendance reasonable if not clinically necessary). This literature highlights that the ED remit of providing life or limb-saving care is key to how staff deliver ED care.”

The point made in the argument above is that the condition affecting the person seeking care is critical to how the staff provide care, as they make a value judgement on the appropriateness of presenting to ED. This means in turn that the reader understanding how the participants were selected (inclusion criteria) as relates to the participants’ presenting complaint and final diagnosis, is crucial to interpreting the results.

The inclusion criteria of patients remains an issue. This is important because it informs on the clinical population interviewed and hence informs the interpretation of results.

It seems that the intention is to recruit participants whose presenting complaint was low back pain. That is people qualified for the study based on the expressed problem on arrival to ED.

This is a distinctly different cohort to including people who have presented to the ED complaining of LBP and after a clinical process have been diagnosed in the ED with musculoskeletal low back pain. In this case the presenting complaint AND diagnosis qualifies them.

That same ED clinical process would differentiate out the cause of the pain and includes infection, visceral pain, kidney infection, kidney stones, ectopic pregnancy, etc…

ED Staff would have a very different approach to an ectopic pregnancy (e.g. appropriate use of ED) compared to standard MSK back pain (e.g. inappropriate).

Greater clarity on exactly how participants were qualified for this study as that relates to their i) presenting complaint and ii) ED diagnosis is still needed.

VERSION 2 - AUTHOR RESPONSE

Requested amendment	
Is there a typo in the first methods paragraph on study design? Should the '3' be there? “Ethical approval was gained from the West of Scotland Research Ethics Committee3 in June 2021 (ref 21/WS/0068).”	Thank you, no. The name of the ethics committee is correctly identified.
This is an argument from the introduction; “This literature argues that key relevant concepts include the primacy of the lifesaving function of the ED; gatekeeping of ED resources (to align with the ED’s primary function); the moral evaluation of patients perceived by staff to have attended illegitimately (those who do not require this acuity of care); and reasonableness (circumstances that make the attendance reasonable if not clinically necessary). This literature highlights that the ED remit of providing life or limb-saving care is key to how staff deliver ED care.”<br>The point made in the argument above is that the condition affecting the person seeking care is critical to how the staff provide care, as they make a value judgement on the appropriateness of presenting to ED. This means in turn that the reader understanding how the participants were selected (inclusion criteria) as relates to the participants’ presenting complaint and final diagnosis, is crucial to interpreting the results.<br>The inclusion criteria of patients remains an issue. This is important because it informs on the clinical population interviewed and hence informs the interpretation of results.<br>It seems that the intention is to recruit participants whose presenting complaint was low back pain. That is people qualified for the study based on the expressed problem on arrival to ED.	Page 6, second paragraph of section Setting, participants and recruitment<br>Has been amended to further clarify the inclusion criteria, including the addition of the words: (no information was sought from the ED team relating to participants’ presentations or management).

This is a distinctly different cohort to including people who have presented to the ED complaining of LBP and after a clinical process have been diagnosed in the ED with musculoskeletal low back pain. In this case the presenting complaint AND diagnosis qualifies them.<br>That same ED clinical process would differentiate out the cause of the pain and includes infection, visceral pain, kidney infection, kidney stones, ectopic pregnancy, etc...<br>ED Staff would have a very different approach to an ectopic pregnancy (e.g. appropriate use of ED) compared to standard MSK back pain (e.g. inappropriate).<br>Greater clarity on exactly how participants were qualified for this study as that relates to their i) presenting complaint and ii) ED diagnosis is still needed.<br><br>Reviewer: 2	

VERSION 3 - REVIEW

**Reviewer**	**2**
**Name**	**Truter, Piers**
**Affiliation**	**The University of Notre Dame Australia, School of Health Sciences**
**Date**	**03-Mar-2025**
**COI**	

Thank you for the revision which makes clear the participant population.

The process of choosing participants based on their description of their presenting complaint introduces heterogeneity in the study population. This may mean that some participants received different care because of the 'appropriateness' of their final diagnosis. Consider updating the limitations section to reflect this.

VERSION 3 - AUTHOR RESPONSE

Comment	Response
Reviewer 2 Piers Truter:<br>The process of choosing participants based on their description of their presenting complaint introduces heterogeneity in the study population. This may mean that some participants received different care because of the 'appropriateness' of their final diagnosis. Consider updating the limitations section to reflect this.	Thank you. As detailed in the methods section and aligning with the population who attend ED, we aimed to include variation in the sample, including a range of LBP presentations (including those for who ED care was and was not likely to be clinically necessary).<br>We considered that the lead author interpreting participants' LBP presentation based on participants' descriptions of their presentation and the care received provided 'good enough' information as we were not stratifying results based on this information and were just seeking to achieve variation in LBP characteristics. We have considered this, but as we were only using this to optimise diversity in the sample, we do not perceive this to be a methodological limitation and thus, have not added it to the limitations.<br>This study provides an analysis of the cultures perceived by patients to inform their experiences of care. Aligning with your previous comments, and as detailed in the analysis, we recognise that important non-clinical reasons inform people's decision to attend the ED, including barriers to accessing healthcare. Recognising that these issues disproportionately affect those with health inequity characteristics, such as living in postcodes that are relatively socially deprived, affirms the importance of all ED care being ethical, dignified and (appropriately) comprehensive, whatever the clinical presentation
